# Footprints in the Sno: investigating the cellular and molecular mechanisms of SNORD116

**DOI:** 10.1098/rsob.240371

**Published:** 2025-03-19

**Authors:** Terri L. Holmes, Alzbeta Chabronova, Chris Denning, Victoria James, Mandy J. Peffers, James G. W. Smith

**Affiliations:** ^1^ Centre for Metabolic Health, Norwich Medical School, University of East Anglia, Norwich, Norfolk NR4 7UQ, UK; ^2^ Department of Musculoskeletal Ageing Science, University of Liverpool, Liverpool, UK; ^3^ Department of Stem Cell Biology, University of Nottingham, Nottingham, UK; ^4^ School of Veterinary Medicine and Science, University of Nottingham, Nottingham, UK

**Keywords:** SNORD116, snoRNA, metabolism, cell signalling, Prader–Willi syndrome

## Abstract

The small nucleolar RNA (snoRNA) SNORD116 is a small non-coding RNA of interest across multiple biomedical fields of research. Much of the investigation into SNORD116 has been undertaken in the context of the congenital disease Prader–Willi syndrome, wherein SNORD116 expression is lost. However, emerging evidence indicates wider roles in various disease and tissue contexts such as cellular growth, metabolism and signalling. Nevertheless, a conclusive mechanism of action for SNORD116 remains to be established. Here, we review the key findings from these investigations, with the aim of identifying common elements from which to elucidate potential targets and mechanisms of SNORD116. A key recurring element identified is disruption to the insulin/IGF-1 and PI3K/mTOR signalling pathways, contributing to many of the phenotypes associated with SNORD116 modulation explored in this review.

**Figure:**
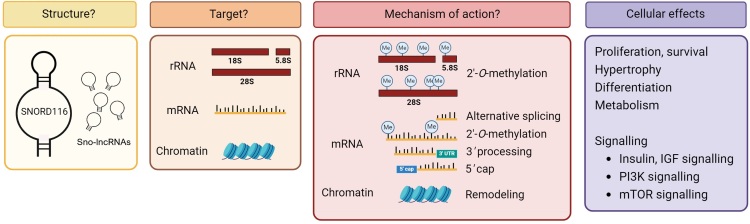

## 1. Introduction

While 40% of the human genome is predicted to be transcribed into RNA, only 2% of the transcriptome is translated into protein [1]. Recent advancements in sequencing and bioinformatic technologies have promoted rapid growth in the field of non-coding RNA (ncRNA), from which small nucleolar RNA (snoRNA) has emerged. Research across various biomedical fields, from cancer to cardiovascular disease, has implicated snoRNAs in disease development and pathology [2–5].

The first snoRNAs identified—U3, U8 and U13—were found in the nucleolus [6–8], hence their nomenclature. However, subsequent studies have demonstrated snoRNAs localize to multiple different cellular and extracellular regions [5,9,10]. SnoRNAs are classified according to their sequence structure; those containing C (RUGAUGA) and D (CUGA) box sequence motifs are referred to as box C/D snoRNAs or SNORDs, and those with H (ANANNA) and ACA sequences are box H/ACA snoRNAs or SNORAs [11].

Canonically, SNORDs enable 2’-*O*-methylation of target ribosomal RNA (rRNA), whereas SNORAs facilitate pseudouridylation of rRNA. Both classes of snoRNAs form ribonucleoprotein complexes with distinct enzymes and proteins to catalyse their respective chemical reactions and hybridize to their target at specific sites within the snoRNA. Currently, there are over 2000 individual entries in the online snoRNA database snoDB [12], which characterizes each snoRNA by type (C/D box or H/ACA box), host gene and target (if known). However, only ~570 entries list rRNA as a target, indicating the majority of identified snoRNAs function in a non-canonical manner. Many snoRNAs have been found to target other types of RNA such as messenger RNA (mRNA) and transfer RNA (tRNA) [13], while some have demonstrated engagement in pathways such as alternative splicing [14,15], alternative polyadenylation [16] and direct protein-binding [17,18].

Almost 1400 of the 2000 snoRNAs listed in snoDB have no specified target and are therefore classed as ‘orphan’ snoRNAs. These snoRNAs present an intriguing, yet challenging topic of study, as there are numerous potential targets, pathways and mechanisms of action to be explored. Moreover, a recent study conducted using snoRNA enriched kethoxal assisted RNA–RNA interaction sequencing (snoKARR-seq) uncovered over 1000 novel snoRNA–RNA interactions, the majority of which do not overlap with known targeting sites [19]. In essence, this demonstrates that there is much still poorly understood regarding how snoRNAs function.

SNORD116 (formally known as HBII-85) is an orphan snoRNA of particular interest, as it lies at the centre of multiple complex and heterogeneous diseases. SNORD116 is most extensively studied in the context of Prader–Willi syndrome (PWS), a congenital disease associated with loss of SNORD116 expression. However, SNORD116 has also been implicated in other diseases including cancer [20–24], cardiovascular disease [25,26] and osteoarthritis [27]. This review summarizes the findings discovered so far in the pursuit of identifying the target(s) and mechanism(s) of SNORD116, highlighting underlying themes recurring across various disease and tissue contexts in which SNORD116 has been investigated.

## 2. Sequence, structure and transcription of SNORD116

**Figure 1.**
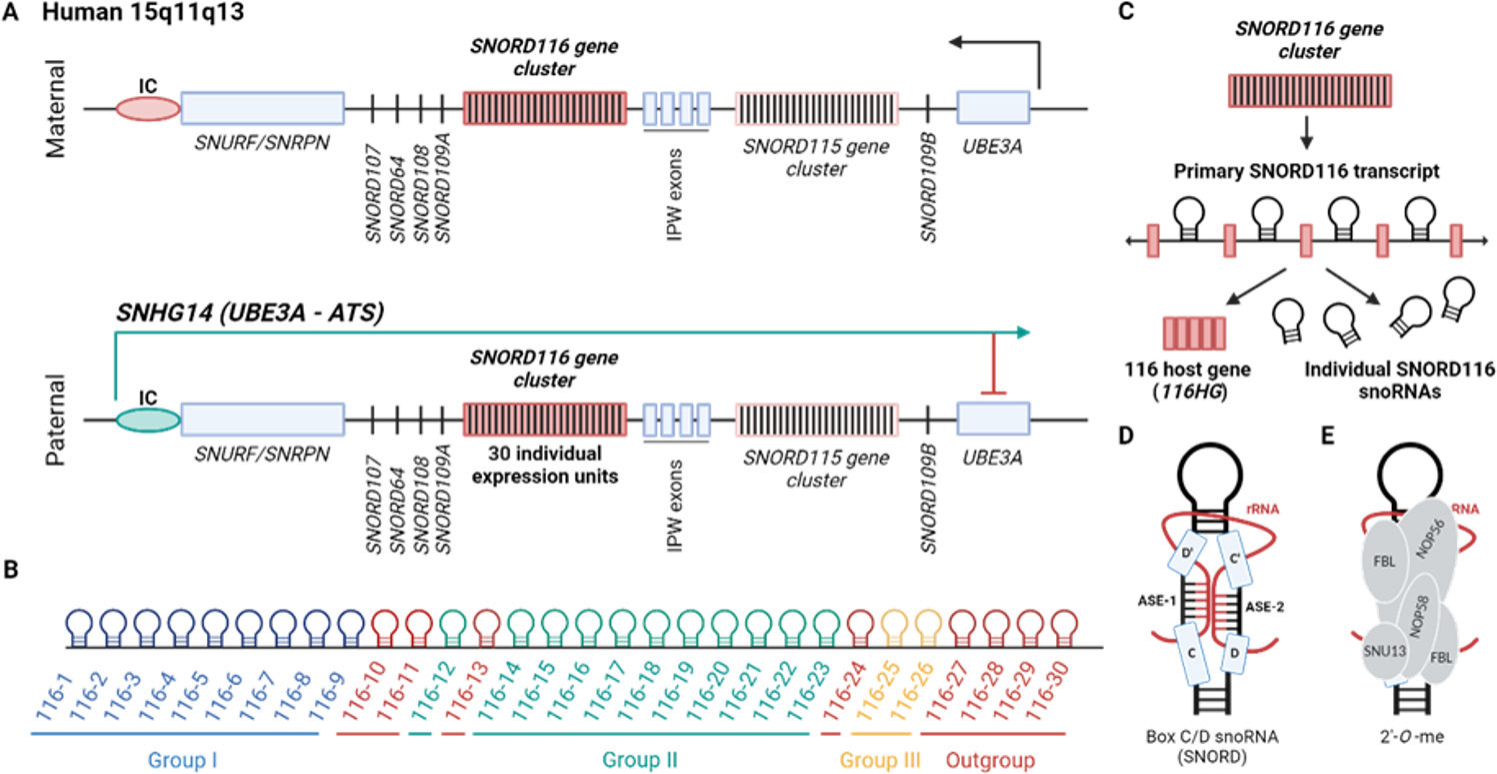
Locus, sequence and structure of SNORD116. (A) The structure of the human *SNHG14* (*UBE3A-ATS*) host gene. The *SNHG14* gene is located on chromosome 15 within the 15q11-q13 locus. A paternally expressed *SNHG14* transcriptional unit is initiated from the unmethylated imprinting control (IC, depicted in green) region and generates a long noncoding transcript which includes SNURF-SNRPN, IPW and UBE3A in the antisense orientation (therefore named *UBE3A-ATS*). The introns of *SNHG14* harbour several snoRNAs, including the *SNORD116* and *SNORD115* gene clusters as well as *SNORD107, SNORD64, SNORD108, SNORD109A* and *SNORD109B*. (B) A schematic of individual SNORD116 copies in humans and their classification according to Baldini *et al*. [28]. (C) A schematic of the SNORD116 gene cluster, its expression and processing. The SNORD116 gene cluster is transcribed into a primary transcript, which is further processed into *116HG (SNORD116 host gene*) and individual SNORD116 snoRNAs. *116HG* is a long noncoding RNA (lncRNA) derived from the spliced exons of the SNORD116 primary transcript. (D) Secondary structure of a canonical box C/D snoRNA (SNORD). It contains the conserved C, C’, D, D’ boxes, and antisense elements (ASE-1, ASE-2) complementary to the target rRNA (ribosomal RNA). (E) The architecture of the canonical box C/D snoRNP complex. SNORDs associate with NOP56, NOP58, SNU13 and methyltransferase Fibrillarin (FBL) which catalyses the 2’-hydroxy methylation (2’-*O*-methylation) of the target rRNA. This figure was created in BioRender.com.

In humans, the SNORD116 gene cluster is located on chromosome 15 at the 15q11-q13 locus. It lies within the intron of the parentally imprinted snoRNA host gene 14 (*SNHG14*, also called *LNCAT* or *UBE3A-ATS*, or *SNURF-SNRPN-sense/UBE3A-antisense transcription unit*; figure 1) [29]. *SNHG14* transcription is initiated from the U exons upstream of the imprinting control (IC) region [30,31]. This region is imprinted, making it functionally haploid. Only the paternal allele is expressed; on the maternally inherited copy of chromosome 15, this region is methylated and therefore silenced [32,33].

The SNORD116 gene cluster consists of several copies of individual SNORD116 box C/D snoRNAs [28,29,34]. In humans, the cluster comprises 30 paralogues, which can be grouped according to their sequence similarity (figure 1; table 1). Historically, SNORD116 has been organized into three groups: Group I: SNORD116-1 to SNORD116-9; Group II: SNORD116-10 to SNORD116-24 and Group III: SNORD116-25 to SNORD116-30 [29,35]. However, a recent analysis has suggested the introduction of a fourth group termed the ‘outgroup’, which contains SNORD116-10, SNORD116-11, SNORD116-13 and SNORD116-27 to SNORD116-30, due to the reduced homology and expression levels of these paralogues [28]. SNORD116 is classed as a box C/D snoRNA due to the presence of specific conserved C and D sequence motifs [11]. Box C/D snoRNAs typically contain two of each motif, with the second denoted with a prime symbol (C′ and D′). Between these motifs are the antisense elements (ASEs), which are unique to each snoRNA and facilitate the binding of the snoRNA to its target via base pairing as depicted in figure 1D.

**Table 1.** Sequence information for each human SNORD116 paralogue. The box C, C’, D and D’ motifs are highlighted in yellow, green, blue and pink, respectively. Group I is shaded in blue, group II in green and group III in red. ‘Outgroup’ paralogues are denoted with an asterisk.

Ensembl ref.	paralogue	SNORD116 exonic sequence	
ENSG00000207063	SNORD116-1	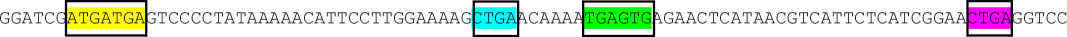	
ENSG00000207001	SNORD116-2	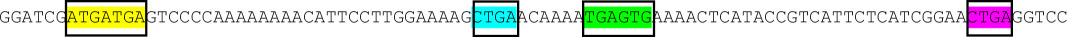	
ENSG00000207014	SNORD116-3	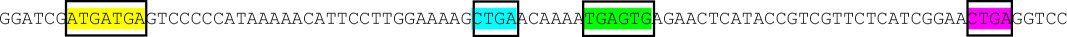	
ENSG00000275529	SNORD116-4	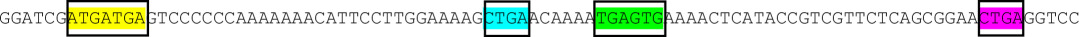	
ENSG00000207191	SNORD116-5	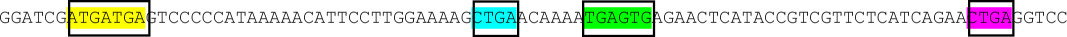	
ENSG00000207442	SNORD116-6	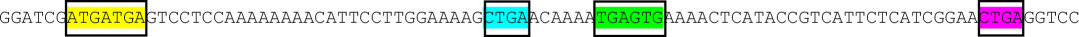	
ENSG00000207133	SNORD116-7	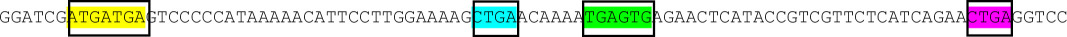	
ENSG00000207093	SNORD116-8	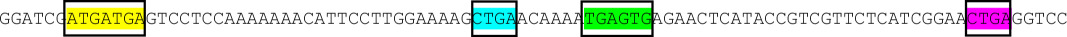	
ENSG00000206727	SNORD116-9	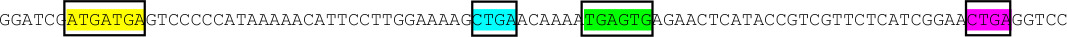	
ENSG00000200661	SNORD116-10*	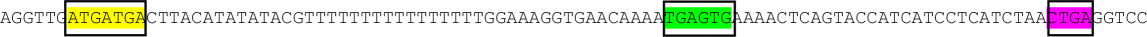	
ENSG00000206609	SNORD116-11*	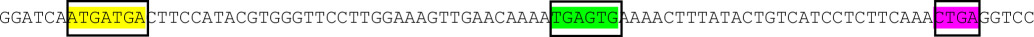	
ENSG00000207197	SNORD116-12	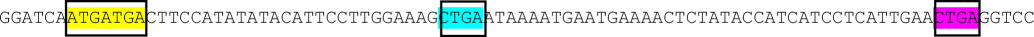	
ENSG00000207137	SNORD116-13*	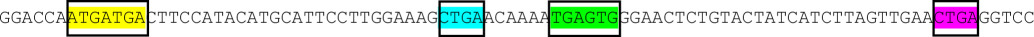	
ENSG00000206621	SNORD116-14	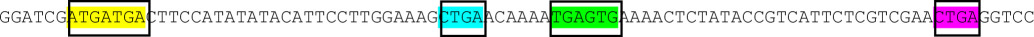	
ENSG00000207174	SNORD116-15	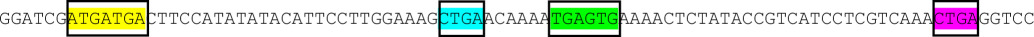	
ENSG00000207263	SNORD116-16	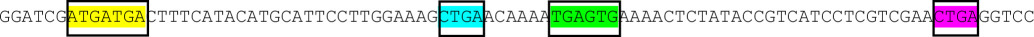	
ENSG00000206656	SNORD116-17	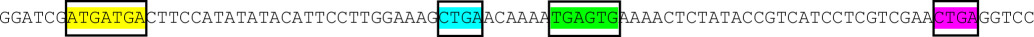	
ENSG00000206688	SNORD116-18	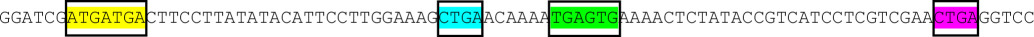	
ENSG00000207460	SNORD116-19	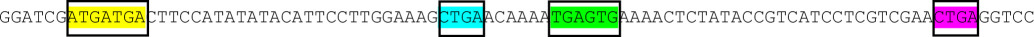	
ENSG00000278715	SNORD116-20	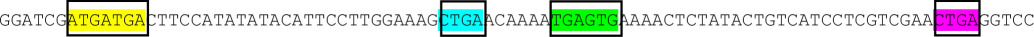	
ENSG00000277785	SNORD116-21	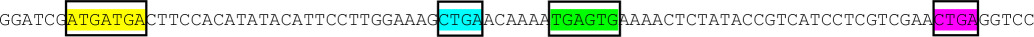	
ENSG00000275127	SNORD116-22	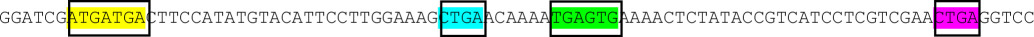	
ENSG00000207375	SNORD116-23	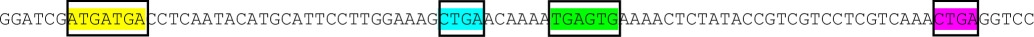	
ENSG00000207279	SNORD116-24	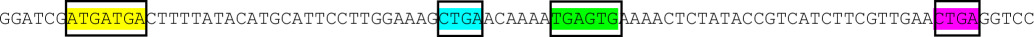	
ENSG00000252326	SNORD116-25	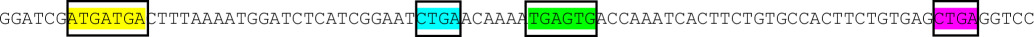	
ENSG00000251815	SNORD116-26	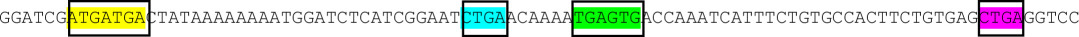	
ENSG00000251896	SNORD116-27*	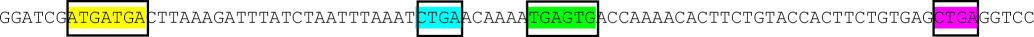	
ENSG00000278123	SNORD116-28*	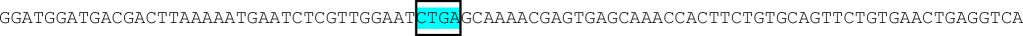	
ENSG00000207245	SNORD116-29*	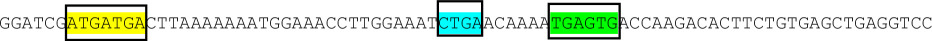	
ENSG00000252277	SNORD116-30*	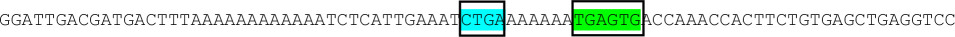	

**Figure 2.**
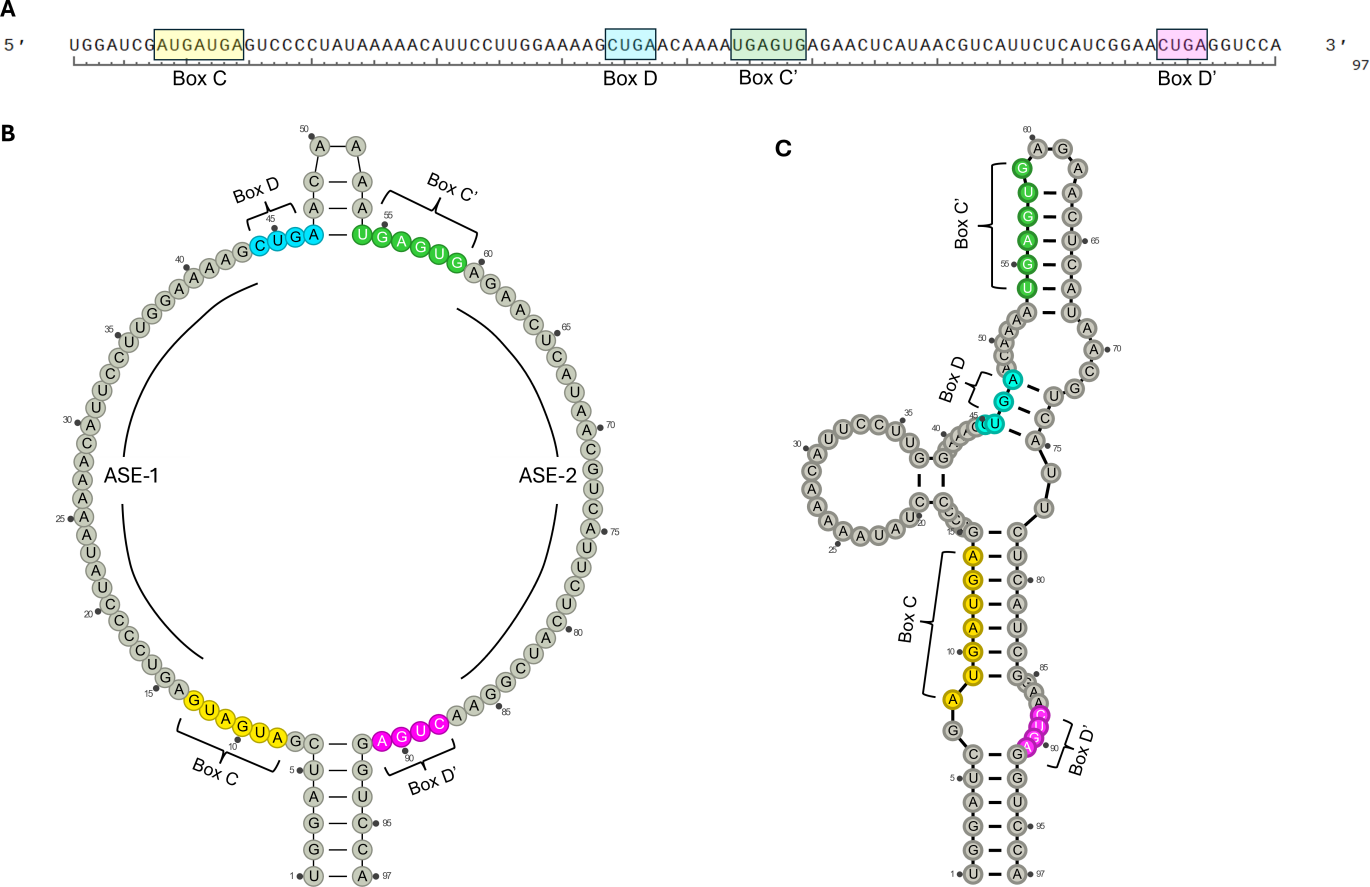
Secondary structure of SNORD116. (A) Sequence of SNORD116-1 with C, C’, D and D’ motifs highlighted in yellow, green, blue and pink, respectively. Generated using SnapGene software (https://www.snapgene.com/). (B) Structure of SNORD116-1 in a canonical box C/D snoRNA form, with a central open structure for snoRNP complex formation. (C) Minimum free energy predicted structure for SNORD116-1, calculated using the RNAfold web server [36]. RNA structures generated using RNArtist (https://github.com/fjossinet/RNArtist).

Nevertheless, no canonical or non-canonical target has been conclusively identified for SNORD116 so far, therefore conferring its status as an ‘orphan’ snoRNA. Furthermore, SNORD116 is not a ‘typical’ representative of the box C/D snoRNA family. For one, the C′-box of SNORD116 snoRNAs (TGAGTG) differs considerably from the consensus (TGATGA) (see table 1) [11]. Second, the sequences of the ASEs, ASE-1 and -2, located upstream of the D′-box and D-box, respectively, vary significantly across the three SNORD116 groups [29]. Third, canonical SNORDs are generally predicted to form a stem-loop structure with a kink-turn and internal base pairing forming within the sequence downstream of the D’ box and upstream of the C’ box, as figure 2 [5,37–41]. However, in SNORD116, the sequence between the D′- and C′-boxes (5′-ACAAAA−3′) does not have the ability to form a stem-loop typical in other SNORDs, which may in turn impact the functionality of the ASEs (figure 2) [42]. However, there is strong evolutionary conservation within a subset of sequences in ASE-1, which implies its importance [28]. Finally, although localization within an intronic region of a host gene is typical for snoRNAs, the highly repetitive gene organization of SNORD116 (and its close neighbours SNORD115 and SNORD114) is less common and may have functional implications. Overall, the various peculiarities of SNORD116 suggest it may not function in a canonical manner compared with other more ‘standard’ box C/D snoRNAs.

During transcription, the SNORD116 gene cluster is transcribed into a SNORD116 primary transcript, which is further processed to generate the SNORD116 host gene (*116HG*) ncRNA and individual SNORD116 snoRNAs [43] (see figure 1). SNORD116 can also be processed into snoRNA-capped long non-coding RNAs (sno-lncRNAs), an unusual class of intron-containing ncRNAs characterized by the presence of a SNORD116 sequence at both ends [44]. During exonucleolytic trimming, the sequences between the snoRNAs are not degraded, leading to the accumulation of lncRNAs flanked by SNORD116 sequences but lacking 5' caps and 3' poly(A) tails. Five sno-lncRNA species were identified: sno-lncRNA1 (SNORD116-6/7), sno-lncRNA2 (SNORD116-13/14), sno-lncRNA3 (SNORD116-18/19), sno-lncRNA4 (SNORD116-20/21) and sno-lncRNA5 (SNORD116-26/27) [45]. It should be noted, however, that while sno-lncRNAs are highly expressed in human and rhesus monkeys, they were not detected in mouse [46], indicating that these sno-lncRNAs may have evolved later to perform species-specific functions. It was also proposed that SNORD116 might be processed to shorter RNA species called psnoRNAs [47], although convincing experimental evidence supporting the existence of these putative psnoRNAs is missing [48].

## 3. Models for SNORD116

Various models have been developed to investigate the biological role of SNORD116. Many focus on the congenital disease PWS, wherein patients do not express SNORD116. The majority of PWS patients harbour large genetic deletions on the paternal 15q11-q13 region, whereas 20−30% have uniparental disomy of the repressed maternal chromosome, and 3% have mutations within the IC region [33,49]. Clinical studies have identified a minimal critical region for PWS which is centred around the SNORD116 gene cluster, implying its cruciality in the development of PWS [50,51]. Nevertheless, the mechanism of action of SNORD116 in PWS is not yet understood. Presenting in early infancy, PWS patients experience abnormalities in a range of biological systems from appetite control to sleep regulation. Motor, language and cognitive development are generally delayed, and most patients suffer with growth hormone deficiency and infertility [52]. Metabolic dysfunction, increased fat deposition and hyperphagia contribute to PWS being the leading genetic cause of obesity in children [53,54]. Respiratory failure and cardiopulmonary disorders are common causes of death in PWS patients, and life expectancy does not generally extend beyond the fourth decade [54]. Much of the academic interest surrounding SNORD116 focuses on its role in PWS, and this impacts the types of models developed. Many are neurological in nature, as behaviour and cognition are key components of the disease. However, SNORD116 exhibits widespread expression throughout the human body, and contemporary research has seen notable development of other model systems and tissues.

### 3.1. Mouse models

Although SNORD116 orthologues have been found in multiple species within the class Mammalia [35], *Mus musculus* is the most typical animal modelling system in SNORD116 research. A comprehensive summary of all existing mouse models of PWS has been reviewed [55]. Multiple groups have tried to recapitulate PWS in the mouse by inducing a chromosomal deletion corresponding to that occurring in humans, often resulting in postnatal lethality [56]. Later models were developed wherein the knockout (KO) was restricted to the Snord116 cluster [57,58]. This resulted in mice that were significantly smaller than their wild-type littermates, remaining so into adulthood (> 1 yr). Postnatal lethality was uncommon, although the mice exhibited motor learning difficulties and increased anxiety [58]. Importantly, Snord116 KO mouse models failed to recapitulate key phenotypes of PWS such as obesity and infertility [55]. This may be due to divergent tissue expression patterns between mice and humans [59]. Mice exhibit lower expression of Snord116 outside the brain, for instance in gonadal tissue [60,61], which may explain why Snord116 KO mice do not exhibit infertility as PWS patients do. Also, the mouse paralogues of Snord116 are highly similar in sequence to the human group I SNORD116 paralogues but share less homology with the group II and III paralogues. These latter groups may have unique functions and contribute to phenotypes that are specific to humans. For these and other reasons, human alternatives to the mouse models have been developed.

### 3.2. Cell models

Loss of SNORD116 in humans has been investigated through the analysis of primary human tissue isolated from PWS patient samples. Due to the predominant cognitive and behavioural phenotypes seen in PWS, samples are often taken from brain tissues such as the hypothalamus [62,63]. However, other tissue types have been studied including whole blood [64], saliva [65] and adipose tissue [66]. Primary human tissue offers invaluable biological insight but is difficult to obtain in large quantities and is subject to variation between individuals.

As an alternative, SNORD116 expression has been investigated and manipulated in various immortalized human cell lines such as human embryonic kidney cells [67], HeLa cells [28] and Lund human mesencephalic (LUHMES) cells [68]. Now with the development of human induced pluripotent stem cell (hiPSC) technology, a wide variety of tissue types can be developed *in vitro* with relative ease. The first PWS iPSC lines were generated in 2010 through the viral transduction of fibroblasts from patients with PWS [69,70]. Since then, the Foundation for Prader–Willi Research have established a PWS biobank from which hiPSC lines can be obtained (https://www.fpwr.org/ipsc-biobank). With this steadily increasing bank of resources, researchers now have a more diverse range of cell models with which to investigate the role and target(s) of SNORD116 in the body.

## 4. Functional effects of SNORD116

This section will explore the different functional phenotypes that arise upon SNORD116 manipulation, summarized in table 2.

**Table 2.** Functional phenotypes associated with dysregulated SNORD116 expression. AD Alzheimer’s disease, CLL chronic lymphatic leukaemia, NSCLC non-small cell lung cancer, CRC colorectal cancer, HCC hepatocellular carcinoma, AML acute myeloid leukaemia, AdMSC adipose-derived mesenchymal stem cell, WAT white adipose tissue, iPSC induced pluripotent stem cell, OA osteoarthritis, RER respiratory exchange ratio, KO knockout, KD knockdown.

phenotype or disease state	tissue context	SNORD116 expression status	reference(s)
reduced proliferation, increased apoptosis	neuroblastoma cells	↓ KO	[62]
AD-associated neurodegeneration	patient serum	↑ upregulated	[71]
cancer	hypoxic glioma cells	↑ upregulated	[72]
	CLL	↑ upregulated	[20]
	NSCLC	↓ downregulated	[21,73,74]
	CRC	↓ downregulated	[22]
	HCC	↓ downregulated	[23]
	AML	↓ downregulated	[24]
increased proliferation	AdMSC	↓ KD	[66]
increased hypertrophy, increased apoptosis	primary subcutaneous WAT	↓ KO (PWS)	[66]
increased physiological hypertrophy	mouse epicardial cells	↑ upregulated	[25]
increased physiological and pathological hypertrophy	human iPSC-cardiomyocytes	↑ upregulated	[26]
OA-associated hypertrophy	primary human chondrocytes	↑ upregulated	[27]
reduced mitochondrial function	primary human fibroblasts	↓ KO (PWS)	[75]
reduced RER	mice	↓ KO	[76]
reduced metabolic gene expression, reduced mitochondrial density, reduced oxygen consumption	primary human adipocytes	↓ KO (PWS)	[66]

### 4.1. Cell proliferation, differentiation and survival

Hyperplasia (growth through cell division) is a crucial component of normal development and homeostasis in many tissues. As cells differentiate their proliferative capacity changes, often becoming less proliferative, particularly in highly specialized cell types such as neurones [77]. Cell turnover is also an essential process in healthy tissue biology, although increased apoptosis is usually an indicator of stress or disease [78]. Evidence suggests that SNORD116 affects these processes in multiple systems and tissues.

Transcriptomic analysis of four tissue samples from the hypothalamus of PWS patients exhibited downregulation of neurone development genes and upregulation in inflammatory and apoptotic genes [62]. Several cell cycle genes were dysregulated including *CDKN1A*, *CDK2* and *TP53*, indicating that loss of SNORD116 may impact cell cycle in hypothalamic tissue. CRISPR-Cas9 was used to engineer a SNORD116 KO human neuroblastoma cell line to investigate how SNORD116 affects neural development and proliferation. An EdU (5-ethynyl-2′-deoxyuridine) incorporation assay identified SNORD116 KO cells had a reduced capacity for proliferation compared to WT, in addition to reduced viability (as measured by fluorescence-activated cell sorting) [62]. The results of this study suggest that loss of SNORD116 may hinder the proliferation in developing neurones, which may impact their maturation and functionality.

In another neural model, SNORD116 expression was increased fivefold during the differentiation of LUHMES cells from embryonic neural progenitors towards polarized dopaminergic neurons [68]. Upon CRSIPR editing, SNORD116 KO did not prevent differentiation but did cause dysregulated gene expression, with the highest number of differentially expressed genes (> 500 DEGs) seen at the later time point (day 15). The majority of DEGs were downregulated in response to SNORD116 KO, indicating that SNORD116 expression may be required in the activation of various gene networks important in later development. Pathway analysis indicated notable dysregulation in cell surface receptor signalling, developmental processes and extracellular matrix cytoskeletal organization, pathways crucial to developmental signalling. Comparison of WT and KO day 15 LUHMES cells with published datasets indicated that the SNORD116 KO expression profile shared a greater overlap with mature neurones compared to WT [68]. Although this assessment is highly subjective, it indicates SNORD116 may have a regulatory role in neuronal maturation. Interestingly, the WT LUHMES cells exhibited greater enrichment of cell cycle-associated genes, supporting findings from previous studies in neuroblastoma cells that loss of SNORD116 correlates negatively with proliferation [62].

SNORD116 has also been investigated in other neural contexts. Alzheimer’s disease (AD) is a neurodegenerative disorder involving the toxic build-up of abnormal protein deposits leading to synaptic destruction and neurone death [79]. PWS patients have been shown to have increased brain age and early onset atrophy, with a brain age gap approximately 9 years older than their chronological age [80]. An analysis on AD mouse models found that Snord116 KO mice exhibited an expression profile that overlapped with AD models [81]. In a study on 23 AD patients versus 16 healthy controls, extracellular vesicles (EVs) were isolated from patient serum and their content analysed using small RNA sequencing [71]. SNORD116 levels were elevated approximately two-fold in the AD EVs. Although the scope of the study only proposed SNORD116 as a potential biomarker of early AD and did not explore the mechanistic causes for this finding, the data could further support a link between SNORD116 signalling and neuronal death.

In addition to neural cells, other more proliferative cell types have also been investigated, such as adipocytes and their progenitors. The uncontrolled weight gain characteristic in PWS has been attributed to lack of appetite control. However, even when compared to non-PWS BMI-matched controls, PWS patients display abnormally expanded total fat mass and reduced muscle mass [82,83]. This indicates cellular dysfunction, possibly in cell proliferation and growth pathways causing abnormal fat expansion.

Transcriptomic analysis of adipose-derived mesenchymal stem cells (AdMSCs) isolated from PWS, and control samples revealed several cell cycle-associated pathways were significantly dysregulated in PWS [66]. Furthermore, an EdU incorporation assay indicated that the PWS AdMSCs had a greater proliferative capacity compared to controls. In agreement with these findings, a SNORD116 knockdown performed in WT AdMSCs also caused an increase in the percentage of EdU-positive cells [66]. As such, in contrast to neural progenitors, loss of SNORD116 appears to have had a pro-proliferative effect on adipose mesenchymal stem cells [66]. These findings suggest that SNORD116 engages with cell proliferation machinery in a cell-dependent manner and may interact with other signalling pathways that affect cell division.

SNORD116 is also dysregulated in multiple forms of cancer, potentiating a role in cancer proliferation and survival. In a study on glioma cells, SNORD116-21 was upregulated 10-fold in response to hypoxic glioma exosome signalling [72]. The hypoxic exosomes also promoted growth of endothelial progenitor cells, indicating that the signalling promoted proliferation and survival alongside upregulating SNORD116. In chronic lymphocytic leukaemia (CCL), SNORD116 was upregulated relative to healthy B Cells [20]. However, in studies investigating non-small cell lung cancer [21,73,74], colorectal cancer [22], hepatocellular carcinoma [23] and acute myeloid leukaemia [24], SNORD116 was downregulated compared to controls. As seen in SNORD116 KO studies, the relationship between SNORD116 and cell growth appears cell type-specific and is probably impacted by the tissue environment.

### 4.2. Hypertrophy

Cellular hypertrophy and cell division are similar processes and indeed are often activated by the same signalling stimuli [84]. Hypertrophy is the process of cell growth without division, whereby the cell increases in size, volume and content but does not enter M-phase or undergo division [85]. Both cell division and hypertrophy are important during and after development, and cellular maturation is often characterized by a change in the balance between the two [86–88].

Normal white adipose tissue (WAT) expands more through hyperplasia than hypertrophy [89], but this process is dysregulated in PWS [49,65,90]. The loss of SNORD116 caused increased hyperplasia in AdMSCs [66]. However, primary subcutaneous WAT from non-obese PWS children and healthy age-matched controls revealed PWS adipocytes to be significantly enlarged and fewer in number [66]. Unlike in the adipocyte progenitor cells, Ki67 immunostaining revealed no difference in mature adipocyte proliferation between PWS and controls. However, similar to the SNORD116 KO neuroblastoma cells [62], PWS adipocytes did exhibit increased apoptosis, which potentially accounts for the overall reduced cell number in the PWS samples [66]. Together these results indicate SNORD116 may regulate hyperplasic growth during adipocyte development, and later hypertrophic growth in mature adipocytes.

Another potential link between SNORD116 and hypertrophy was detected in cardiac tissue. In a mouse model of physiological cardiac hypertrophy, SNORD116 was upregulated ~7-fold in epicardial cells [25], indicating that it may be involved in pro-hypertrophic signalling that occurs within the epicardium. Several Forkhead box (FOX) transcription factors were also upregulated including FOXG1, FOXA3 and FOXS1 [25]. In mouse heart tissue, immunostaining revealed exercise-induced cardiac hypertrophy caused an increase in the percentage of FOXG1-expressing epicardial cells. Increased *FOXG1* expression correlated with increased *PCNA* expression, and therefore increased proliferation. Loss of Snord116 did not alter the expression of *FOXG1* in mouse epicardial cells, but loss of *FOXG1* did cause reduced Snord116 expression, and a reduction in epicardial cell proliferation [25]. These results suggest that FOXG1 may regulate epicardial cell proliferation in response to physiological hypertrophy, potentially through modulating Snord116.

SNORD116 was also elevated in a hiPSC-derived cardiomyocyte (hiPSC-CM) model of hypertrophic cardiomyopathy [26]. IPSC-CMs with the c.ACTC1^G301A^ mutation recapitulated the phenotype of HCM through abnormal contractility and Ca^2+^ handling, impaired oxidative phosphorylation, increased arrhythmogenesis and brain natriuretic peptide (BNP) signalling [91]. EV analysis revealed increased SNORD116 packaging in HCM iPSC-CMs in response to increased workload [26]. These results suggest that SNORD116 signalling may play a role in cardiomyocyte hypertrophy.

Hypertrophy is also important outside of the heart, and SNORD116 has also been linked to hypertrophic signalling in cartilage. SNORD116 was found to be elevated in cartilage samples of patients with OA [27]. Chondrocytes isolated from OA patients had elevated expression levels of hypertrophic *COL10A1* and *MMP13*, alongside decreased expression of chondrogenic *COL2A1*. Analysis of snoRNA expression revealed that the hypertrophic chondrocytes from OA cartilage had elevated levels of SNORD116. Furthermore, upon hypertrophic stimulation of primary human articular chondrocytes isolated from non-OA patients, SNORD116 expression increased. Interestingly, comparison of old non-OA cartilage with young non-OA cartilage found a decrease in SNORD116 expression, implying that the upregulation of SNORD116 in OA is associated specifically with chondrocyte hypertrophy as opposed to non-pathological ageing processes [27]. Overall, this study further emphasizes the potential relationship between hypertrophy and SNORD116.

Taken together, these results show that across multiple tissue types, SNORD116 expression appears elevated in response to pro-hypertrophic signalling and may modulate hypertrophy in a cell-stage and cell type-dependent manner.

### 4.3. Metabolism

Metabolism is a key component of cellular developmental biology. The metabolic state of a cell not only reflects its development but also its health [92], as stress and disease can dysregulate metabolism and cause the cell to destabilize [93–95]. Evidence suggests that SNORD116 may impact cells through changes in metabolism.

PWS is a disorder with multiple phenotypes including deregulated metabolism. Demonstrating this, metabolic analysis comparing the respiration of fibroblasts derived from healthy and PWS patients found reduced mitochondrial function in the PWS cells [96]. Further investigation has been carried out in mice, where loss of Snord116 disrupted behaviour and metabolism. Snord116 KO mice exhibited a reduced respiratory exchange ratio (RER), indicating a metabolic shift away from carbohydrate metabolism and towards fatty acid oxidation [76]. Mice are nocturnal and typically exhibit higher RER values during the nighttime, and decreased RER in the day when their activity levels are lower [97,98]. During the daytime, Snord116 KO mice had decreased RER compared to WT, indicating decreased carbohydrate metabolism. The KO mice also had a smaller body weight, despite showing no change in food intake or activity compared to WT, indicating an underlying impediment in energy storage or usage.

Deregulated fat deposition is a damaging phenotype in PWS [99,100]. Comparing PWS adipose tissue to healthy controls within a specialized metabolism-regulating population of adipocytes known as ‘beige adipocytes’, the PWS cells exhibited reduced mRNA and protein expression of key metabolic genes such as peroxisome proliferator-activated receptor alpha (PPARα), fatty acid binding protein 3 (FABP3) and peroxisome proliferator-activated receptor gamma coactivator 1-alpha (PGC-1) [66]. Mitochondrial density and oxygen consumption were suppressed in PWS-differentiating beige adipocytes compared with control cells. Beige adipocyte differentiation was also impeded by SNORD116 knockdown, indicating the metabolic impacts of SNORD116 may also affect developmental pathways (or vice versa). SNORD116 expression increased during beige adipogenesis in WT cells, however upon knockdown the adipocytes exhibited decreased lipid content, reduced mitochondrial density and the downregulation of beige adipocyte markers such as PPARγ, the master regulator of adipogenesis. Furthermore, transcriptomic analysis of AdMSCs isolated from PWS and control samples revealed PPARγ was notably reduced in the PWS cells at both an mRNA and protein level [66]. Overall fatty acid metabolism was impeded by loss of SNORD116, which may contribute to the metabolic phenotypes seen in PWS.

Further evidence of a metabolic role for SNORD116 was found in an embryonic mouse hypothalamic cell line, where overexpression of Snord116 was achieved using a plasmid [41,67]. This caused a ~15-fold increase of Nescient Helix-Loop-Helix 2 (NHLH2), a transcription factor that, when knocked out in mice, caused adult-onset obesity and reduced physical activity [101]. *In silico* RNA: RNA binding analysis software predicted a 20 bp interaction between SNORD116-3 and *Nhlh2* mRNA. SNORD116 overexpression also altered the decay rate of *Nhlh2* mRNA, increasing its stability and therefore increasing the translation of the protein. Another study found *Nhlh2* mRNA was decreased in PWS iPSC-derived neurones [61], further implicating *Nhlh2* mRNA as a target of SNORD116. According to the human protein atlas [102], *Nhlh2* is a transcription factor expressed primarily in brain tissues. Regarding its metabolic role, *Nhlh2*-KO mice displayed phenotypes reflective of PWS including obesity, hypogonadotropic hypogonadism and overall impaired metabolism [101,103]. Additionally, variants in the *Nhlh2* gene have been linked with obesity in humans [104], which together suggest that the reduced NHLH2 activity due to lack of SNORD116 in PWS may contribute to the obesity-related metabolic phenotypes seen in patients.

Evidence across multiple tissue types and model systems revealed SNORD116 to be involved in differentiation, proliferation, hypertrophy and metabolism. These four processes are crucial to cellular developmental biology, and all contribute to the regulation of each other during normal development in the majority of tissue types. Given this evidence, it is therefore likely that the target(s) of SNORD116 exists in the underlying signalling pathways controlling one or all of these phenotypes, and so these signalling pathways must be explored further.

## 5. Signalling pathways

Cellular functions such as proliferation and metabolism are initiated and controlled through cell signalling pathways. There are many examples of snoRNAs participating in signalling mechanisms to affect cell behaviour [2,105–109]. SnoRNAs modulate diverse signalling networks [3,5,110], and SNORD116 is probably no exception. Investigating the signalling pathways affected by SNORD116 is a crucial step to understanding its role in human disease.

### 5.1. Insulin and insulin-like growth factor signalling

Insulin and insulin-like growth factor (IGF) signalling is the primary control mechanism of glucose homeostasis, but it is also important in a wide variety of other functions including lipid metabolism, protein synthesis and apoptosis [111]. Activation of the insulin receptor leads to activation of the Ras/MAPK/ERK signalling cascade, and/or phosphoinositide 3-kinase (PI3K)/Akt/mTOR pathway activation [112]. Cell cycle and survival, glucose transport, metabolism and protein synthesis are generally modulated via the PI3K/Akt pathway, whereas the MAPK pathway controls proliferation and transcription [113]. Studies have shown SNORD116 ablation may alter the cell’s response to insulin, and therefore impact downstream signalling pathways.

Dysregulated insulin signalling is a consistent phenotype of PWS, attributable to growth hormone deficiency and hypoinsulinemia [61,114,115]. Demonstrably, a study of 13 PWS patients (versus 18 non-PWS controls) found that IGF binding protein 7 (IGFBP7) was elevated 1.75-fold [116]. PWS patient-derived iPSC neurones also exhibited elevated gene expression of *IGFBP7* [116]. Similarly, plasma and brain tissue samples from a mouse model of PWS exhibited elevated IGFBP7 levels, although expression in liver and heart tissue was contrastingly decreased compared to WT [116,117].

In another study, primary fibroblasts isolated from PWS patients exhibited a reduced response to insulin compared to controls [118]. When exposed to insulin, control fibroblasts exhibited an increase in nascent protein synthesis, whereas PWS fibroblasts showed no change in response to insulin. Interestingly, prior to insulin exposure, the PWS fibroblasts at baseline exhibited higher levels of nascent protein synthesis relative to controls, which may relate to the apparent lack of response when insulin was introduced. Further analysis revealed that insulin exposure in control fibroblasts leads to increased phosphorylation of p70S6K1, a target of mTOR (mammalian target of rapamycin) that promotes translation [119,120]. However, insulin exposure failed to elicit this response in PWS fibroblasts [118]. The results suggest loss of SNORD116 may cause an impairment in the cell’s response to insulin, and the snoRNA may be important in the propagation of the pathway between activation of the insulin receptor and downstream phosphorylation events. This may explain why in some studies, loss of SNORD116 caused an increase in apoptosis [62,66], as insulin is known to decrease oxidative stress and reduce apoptosis [121]. In the clinic, PWS patients are consistently found to have increased insulin sensitivity compared to non-PWS patients [90,122], which further indicates a potential defect downstream in the pathway caused by loss of SNORD116.

### 5.2. PI3 kinase signalling

Insulin/IGF-1 signalling is an activator of the PI3K/Akt/mTOR signalling pathway; a master regulator of multiple key cellular functions including metabolism, cell proliferation and protein synthesis [123–125]. Evidence suggests SNORD116 may alter PI3K signalling under certain conditions.

ChIRP-seq (Chromatin Isolation by RNA purification) of WT mouse brain samples found several PI3K-related genes enriched for binding to Snord116 including mTOR, transcriptional regulator Creb-binding protein (Crebbp) and imprinted insulin growth factor receptor (Igf2r) [76]. Upon Snord116 KO, mouse cortex tissue showed an increase in levels of mTOR and an increase in phosphorylated S6 ribosomal protein, suggesting SNORD116 may have a suppressive role in mTOR signalling [76].

If SNORD116 is a negative regulator of PI3K signalling, this may account for the apparent downregulation of SNORD116 in some forms of cancer such as AML and lung cancer (see table 2). This may also explain why PWS fibroblasts exhibited a higher level of nascent protein synthesis compared to controls prior to insulin exposure [118], as mTOR activity is associated with increased translation. PI3K signalling is also important in hypertrophic growth, and as discussed in this review, SNORD116 expression has been linked with hypertrophy in multiple cell types. Although interaction between SNORD116 and PI3K signalling is speculative, investigating the relationship between them may be the key to understanding its function.

## 6. Mechanism of action

Understanding how SNORD116 interacts with its target is crucial to understanding the function of SNORD116 in development and disease. Unfortunately, the mechanism of SNORD116 remains as elusive as its target. However, there is incrementing evidence providing some insight into the mechanistic action of SNORD116.

### 6.1. RNA binding

A recent study used snoKARR-seq to identify novel RNA binding interactions of Snord116 expressed in the mouse brain cortex [19]. A total of 32 interactions were identified across different targets including mRNA, lncRNA, tRNA and small nuclear RNA (snRNA) [19]. Interestingly, some of the targets such as *Malat1*, *Meg3* and *Rny3* are associated with cancer [126–129] and therefore may relate to the role of SNORD116 in cell turnover and/or oncogenesis. However, it is important to note that the same study examined snoRNA–RNA interactions in multiple human cell lines such as HepG2, A549 and HEK293T and the majority of interactions uncovered were unique to cell line [19]. This indicates that the environment and cell type in which the snoRNA is expressed plays a significant role in target binding. This is an important consideration when finding a target for SNORD116, and is further evidence that the role of SNORD116 may vary across different tissues.

### 6.2. Methylation

SNORDs function canonically by guiding 2’-*O*-methylation of rRNAs. To catalyse this chemical reaction, SNORDs associate with four evolutionary conserved proteins: Fibrillarin (FBL), SNU13, NOP58, and NOP56, together forming a ribonucleoprotein complex (snoRNP) [11,130]. While SNORDs site-directionally guide 2’-*O*-methylation by base-pairing (via the ASE) with substrate rRNAs, the methyltransferase FBL catalyses the 2’-hydroxyl methylation of the ribose moiety [131,132].

Although SNORD116 does vary slightly in sequence homology with classic box C/D snoRNAs, it seems to be capable of forming a snoRNP complex. Immunoprecipitation experiments of rat brain extracts showed that Snord116 is associated with FBL and NOP58 [133]. Together with the fact that SNORD116 snoRNAs seem able to localize to the nucleolus [134], this suggests that SNORD116 might guide 2’-*O*-methylation and play a role in ribosome biogenesis. RISE, a comprehensive database of the RNA interactome from sequencing experiments, proposes candidate interactions of human SNORD116 snoRNAs with other snoRNAs, mRNAs, and lncRNAs [135]. However, experimental validation confirming these candidate interactions is yet to emerge.

Interestingly, there is evidence that SNORD116 impacts DNA methylation. A study using Snord116 KO mice cortex samples found that loss of SNORD116 disrupted methylation patterns in over 23 000 CpGs [136]. Samples were taken from WT mice at various time points during light and dark hours of the day, and there were over 4000 differentially methylated regions exhibiting diurnal rhythmic methylation. In 97% of these regions, the methylation pattern was lost or shifted in the KO, indicating that SNORD116 is important in diurnal methylation. In mice and humans, diurnal methylation is an important control mechanism within the circadian rhythm which has significant metabolic consequences if disrupted [137]. Sleep disturbance and metabolic dysregulation are common symptoms in PWS patients and PWS mouse models [138,139], which may be due to the disruption of SNORD116 as a modulator of DNA methylation. However, mechanistic evidence of this interaction is yet to be established.

### 6.3. Alternative splicing

Some snoRNAs are known to act non-canonically and influence the alternative splicing of target genes [15,140]. A particularly relevant example is the box C/D snoRNA SNORD115, located just downstream of SNORD116 on human chromosome 15. SNORD115 regulates the expression of *serotonin receptor 2C (5-HT2CR*) by promoting alternative splicing of its pre-mRNA [14]. There are two alternative 5’ splice sites in exon V of *5-HT2CR* pre-mRNA, resulting in two potential isoforms: exon Va and exon Vb. Only mRNAs containing exon Vb encode a functional protein [141]. SNORD115 was shown to promote inclusion of exon Vb by binding to and blocking a silencer located within exon Vb [14,142]. SNORD116 may function in a similar manner and influence the alternative splicing of target genes. However, it is important to note a later study conducted using a Snord115 KO mouse demonstrated no conclusive evidence that SNORD115 can regulate *5-HT2CR* alternative splicing, suggesting this mechanism may be specific to human cells [143], which may also be the case for SNORD116.

In a study in human LUHMES cells, the analysis of pre-mRNAs showed no notable differences in alternative splicing as a result of SNORD116 KO, indicating the snoRNA is unlikely to influence alternative splicing in this context [68]. Interestingly though, proteomics analysis revealed differences in protein abundance relative to mRNA levels in numerous genes, demonstrating post-transcriptional changes propagated by SNORD116 KO.

A study in human embryonic stem cells found evidence of interaction between SNORD116-derived sno-lncRNAs and the splicing factor FOX2 [44]. In addition to identifying multiple predicted binding sites within FOX2, knockdown of the SNORD116-derived sno-lncRNAs resulted in altered alternative splicing of FOX-regulated genes. The authors hypothesize that SNORD116-derived sno-lncRNAs act as a molecular sink controlling FOX2 localization and activity [44].

Bioinformatic analysis has corroborated the role of SNORD116 in alternative splicing. Several groups performed computational target prediction analysis to identify targets for SNORD116. Bazeley *et al*.[42] used snoTARGET to examine possible targets for 27 ASE-2 sequences of human SNORD116 snoRNAs [42]. Fourteen of SNORD116 snoRNAs showed significantly elevated guiding specificity towards exons compared to introns. Moreover, their exonic targets were within genes producing alternatively spliced mRNA isoforms such as *DRF1* and *GTPBP3*, therefore, suggesting that SNORD116 might regulate alternative splicing [42].

More recently, Baldini *et al*. [28] adapted BLAST analysis to compile a list of genes that could hybridize to either ASE-1 or ASE-2 sequences in human and mouse SNORD116 [28,144]. Three mRNAs were shared by the two species: *Dgkk*, Neuroligin 3 (*Nlgn3*) and the round spermatid basic protein 1 like (*Rsbn1l*). The functional analysis evaluating the capacity of SNORD116 to affect the expression of these three candidate mRNA targets in human HeLa S3 cell line suggested that the mRNAs identified in the interaction screen are in indeed robust candidate effectors of SNORD116 function [28]. Nevertheless, a further investigation is necessary to validate these results and uncover the underlying mechanism of action.

In addition to alternative splicing, SNORD116 has been speculated to modulate alternative polyadenylation. When examining a putative interaction site between SNORD116 and *Nhlh2* mRNA, a poly(A) signal was identified around 100 bp downstream [41]. The authors hypothesized SNORD116 may alter the stability of *Nhlh2* mRNA via the poly(A) tail. There is precedence for box C/D snoRNAs regulating mRNA 3' processing, as SNORD50A was found to block a subunit of the cleavage and polyadenylation specificity factor (CPSF), thus promoting the alternative polyadenylation of several genes [16].

## 7. Conclusions and future perspectives

**Figure 3.**
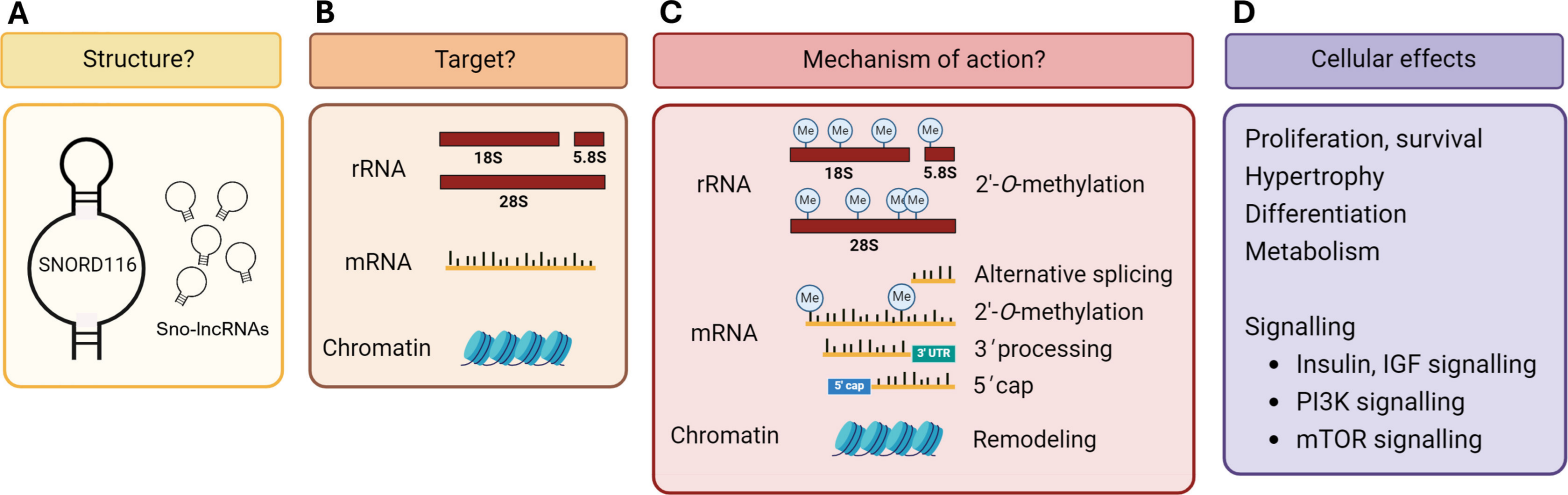
Open questions surrounding SNORD116. (A) SNORD116 is predicted to adopt the classical box C/D formation, although several unique elements within its sequence may affect its ability to form this structure. SNORD116 may also exist as sno-lncRNAs. (B) Canonical box C/D snoRNAs target rRNA, however, there are no known rRNA targets for SNORD116. Other possibilities include mRNA and DNA. (C) Canonical box C/D snoRNAs facilitate 2’-*O*-methylation; however, there is evidence suggesting SNORD116 may affect target splicing or stability. (D) Cellular effects of SNORD116 modulation are cell-type specific but may be a result of dysregulation of common pathways such as insulin/IGF-1 and PI3K/mTOR signalling. This figure was created in BioRender.com

SNORD116 has been widely studied, yet no validated target or mechanism of action has been conclusively determined. Data suggest SNORD116 is involved in multiple pathways and appears to have differing effects depending on the cell type and tissue context in which it is expressed. As outlined in figure 3, there are many open questions surrounding SNORD116 and numerous possibilities regarding its target and mechanism. This review summarized evidence across diverse studies and experimental models and evidenced a variety of different cellular phenotypes associated with SNORD116 expression. The key signalling events underpinning these changes which become disrupted when SNORD116 is downregulated, appear within the insulin/IGF-1 and PI3K/mTOR pathways. However, these events may themselves be consequences of disruption to the primary target of SNORD116, which remains elusive. Determining the function and mechanism of SNORD116 would help in our understanding of numerous pathologies including and beyond PWS, and may reveal new targets in the development of novel disease treatments.

## Data Availability

All DNA sequences were obtained from the Ensembl database; reference codes are provided in table 1.
